# BASIC: BCR assembly from single cells

**DOI:** 10.1093/bioinformatics/btw631

**Published:** 2016-10-02

**Authors:** Stefan Canzar, Karlynn E Neu, Qingming Tang, Patrick C Wilson, Aly A Khan

**Affiliations:** 1 Toyota Technological Institute at Chicago, The University of Chicago, Chicago, IL, USA; 2 Committee on Immunology, The Knapp Center of Lupus and Immunology Research, The University of Chicago, Chicago, IL, USA

## Abstract

**Motivation:**

The B-cell receptor enables individual B cells to identify diverse antigens, including bacterial and viral proteins. While advances in RNA-sequencing (RNA-seq) have enabled high throughput profiling of transcript expression in single cells, the unique task of assembling the full-length heavy and light chain sequences from single cell RNA-seq (scRNA-seq) in B cells has been largely unstudied.

**Results:**

We developed a new software tool, BASIC, which allows investigators to use scRNA-seq for assembling BCR sequences at single-cell resolution. To demonstrate the utility of our software, we subjected nearly 200 single human B cells to scRNA-seq, assembled the full-length heavy and the light chains, and experimentally confirmed these results by using single-cell primer-based nested PCRs and Sanger sequencing.

**Availability and Implementation:**

http://ttic.uchicago.edu/∼aakhan/BASIC

**Supplementary information:**

Supplementary data are available at *Bioinformatics* online.

## 1 Introduction

**Fig. 1:**
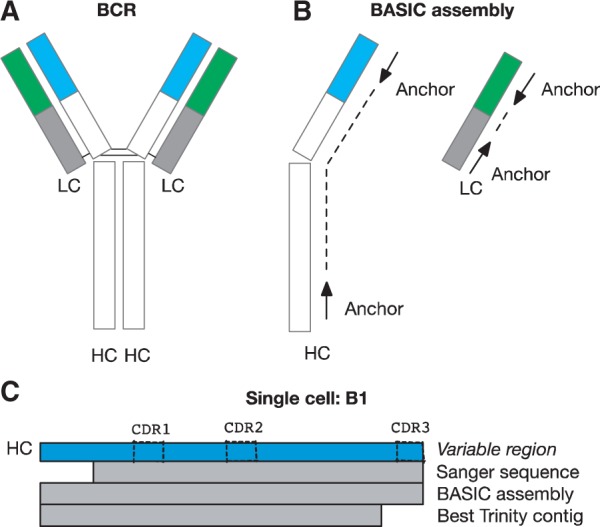
(**A**) The BCR is a large ‘Y’ shaped protein complex composed of two identical heavy chains (HC) and two identical light chains (LC). The complementarity determining regions (CDRs) are those parts of the variable regions that participate in the binding of antigens. (**B**) Anchors are stitched together to assemble the HC and LC. (**C**) Illustration of the HC variable region sequence for single-cell PW1_B1 along with the Sanger sequence, the BASIC assembled sequence, and the best contig reported by Trinity. Note the absence of CDR3 from the Trinity contig, and the BASIC assembly extending past the 5’ PCR primer site used in the Sanger sequence (Color version of this figure is available at *Bioinformatics* online.)

B cells form an important component of the adaptive immune system. They possess the remarkable capacity to recognize antigens through the B-cell receptor (BCR; Fig. 1A), which is generated through a series of somatic rearrangements and mutations that allow for more than 10^18^ possible sequences (Davies *et al.*, 1975; Edelman, 1959; Elhanati *et al.*, 2015). Single-cell RNA-sequencing (scRNA-seq) offers a particularly powerful way to study adaptive immune cells, simultaneously allowing both for capture of quantitative gene expression as well as repertoire sequence information (Eltahla *et al.*, 2016; Stubbington *et al.*, 2016). By coupling gene expression with specific BCRs, exciting opportunities emerge in studying B-cell biology that are not approachable with bulk RNA-seq and repertoire studies, such as characterizing at single-cell resolution the transcriptional heterogeneity underlying BCR affinity maturation and receptor specificity.

The assembly of full-length BCR sequences from scRNA-seq is a non-trivial problem. On the one hand, reference-based assembly methods (Pertea *et al.*, 2015) that rely on an initial alignment of reads to a genome are vulnerable to the somatic rearrangements and mutations present in BCR sequences. On the other hand, *de novo* assembly methods typically require solving a noisy and complex genome-scale assembly task that makes targeted BCR assembly error prone. Thus, the lack of efficient methods for assembling BCR sequences from scRNA-seq data is a major roadblock in advancing B-cell biology.

The BCR is generated, initially, by a process called V(D)J recombination, where a single gene in each of the three categories (V, D and J) is chosen from a pool of possibilities and recombined at the DNA level (Tonegawa, 1983). During V(D)J recombination additional genetic variability is achieved through junctional diversity or the inexact joining of gene segments. The heavy chain is unique in its inclusion of the ‘D’ gene segment, which specifically generates increased genetic diversity in the antigen-recognizing domain. In addition to the extreme diversity introduced by this cell-specific recombination event, BCRs also undergo a process called affinity maturation, where somatic hypermutation introduces random point mutations in the V(D)J region of both heavy and light chains (Rajewsky *et al.*, 1987; Weigert *et al.*, 1970). Finally, the inclusion of different constant regions at the base of the receptor in the heavy chain helps to determine the effector functions when the BCR is secreted in its soluble form as an antibody. All of these elements result in an extremely complex locus.

Here, we developed a novel semi-*de novo* assembly method to determine the full-length sequence of the BCR in single B cells from scRNA-seq data, called BASIC (*B*CR *as*sembly from *si*ngle *c*ells). To demonstrate the utility of our method, we subjected nearly 200 single human B cells to scRNA-seq and assembled full-length heavy and light chains. We then experimentally validated these results by using single-cell PCR and Sanger sequencing, and demonstrate overall high accuracy.

## 2 Results

### 2.1 BASIC algorithm

Full details of the BASIC algorithm can be found in the Supplementary text. Briefly, BASIC performs semi-*de novo* assembly in two stages (Supplementary Fig. S1): Stage 1, BASIC uses known constant and variable regions to identify anchor sequences; Stage 2, BASIC uses these anchors to guide the *de novo* assembly of the BCR.

*Stage* 1. The BASIC software uses a pre-compiled database of known variable (IGHV, IGKV, IGLV) and constant (IGHC, IGKC, IGLC) region sequences in human from IMGT (http://www.imgt.org). The database was indexed using Bowtie2 (Langmead and Salzberg, 2012) into four distinct files that corresponded to different components (IGHV; variable heavy chain, IGHC; constant heavy chain, IGK/LV; variable light chain, IGK/LC; constant light chain). Second, BASIC calls Bowtie2 in order to align scRNA-seq reads from a single B cell to each of the four component index files. Third, for each component, BASIC identifies a short sequence window containing the highest number of aligned reads. These four sequences served as anchors to guide the assembly stage.

*Stage* 2. Next, BASIC performs *de novo* assembly to stitch together the anchor sequences in the heavy and light chains (Fig. 1B). We assume a sequence may overlap either with the forward sequence or the reverse complement sequence of another read. Two reads overlap if the prefix of one sequence equals the suffix of the other sequence (or *vice versa*). Briefly, BASIC extends each anchor iteratively in the 3' direction (one read at a time) until there is either no overlapping read or a repeat is found. Then, each anchor is extended in the 5’ direction in the same way. For each chain, BASIC reports a single sequence if the extended sequence from the variable region anchor is equal to the extended sequence from the constant region anchor. However, if the two sequences did not match, BASIC reports both an extended variable region contig and an extended constant region contig, separately.

The running time of BASIC mostly depends on the length of the sequencing reads and the number of reads. BASIC is implemented in Python and uses multi-core processing to assemble the heavy and light chains simultaneously.

### 2.2 Experimental validation of BASIC assembled sequences

We obtained antibody producing B cells (plasmablasts) by fluorescence-activated cell sorting from the peripheral blood of seven human donors. These samples were subjected to scRNA-seq using an adapted version of the SmartSeq2 protocol (Picelli *et al.*, 2014). Some of these adaptations included testing different pre-amplification PCR cycles (Column B, Supplementary Table S1), and the removal of a small aliquot of amplified full-length cDNA to validate BASIC predictions using nested PCR-based BCR amplification and Sanger sequencing (Smith *et al.*, 2009; Wrammert *et al.*, 2008). The nested PCR utilized a cocktail of primers for all possible V and J genes. Single-cell RNA-seq libraries were then prepared by tagmentation, and single-end (17 samples) or paired-end (174 samples) 50 base-pair sequencing was performed on an Illumina HiSeq2500 machine. In sum, our data set contains 191 single cells, which resulted in 382 V(D)J sequences (Supplementary Table S1).

In 37 situations, our initial nested PCR was unable to amplify the BCR using a cocktail of primers, potentially due to competition between primers preventing amplification or a mutation in the primer-binding site (Column D, Supplementary Table S1). This resulted in a unique opportunity to apply and test BASIC, where we then selected specific gene primers based on the prediction of the BCR from scRNA-seq data. Consequently, we performed a series of secondary PCRs by using specific primers to amplify the BCR based solely on BASIC and successfully recovered all 37 sequences (Column AA, Supplementary Table S1).

#### 2.2.1 BASIC is highly accurate in predicting BCR sequences

BASIC successfully identified V(D)J gene usage as obtained by Sanger sequencing in both heavy and light chains in 370/382 (∼97%) of the sequences. Importantly, BASIC was able to correctly identify the V(D)J sequence across a range of somatic alterations and sequencing depths (Columns M, Y, AJ, Supplementary Table S1).

BASIC failed to correctly assemble 12/382 (or ∼3%) of the immunoglobulin sequences. It stands to reason that sample quality and, in turn, sequencing coverage of the BCR is a major determinant for accurate assembly. While BASIC does not explicitly use pairing information, the additional sequences generated from paired-end sequencing likely provides for greater coverage of the BCR and implicitly facilitates the *de novo* assembly stage in BASIC. Consistent with this, 5/12 of the incorrect predictions were obtained from single-end sequencing data. Taken together, BASIC correctly assembles full-length BCR sequences across a range of sequencing and experimental conditions including somatic mutations, sequencing depths and pre-amplification PCR cycles.

##### 2.2.2 BASIC harnesses anchors to guide accurate BCR assembly

We sought to determine the importance of using anchors to guide assembly in our semi-*de novo* approach. We assembled all 191 scRNA-seq samples using Trinity, a state-of-the-art, purely *de novo* approach (Grabherr *et al.*, 2011). Because a limitation in using a purely *de novo*-based method is identifying BCR related sequences among the hundreds of thousands of contigs assembled, we assumed we had an oracle to tell us the single best contig by aligning all of Trinity's contigs to the true reference BCR sequence using BLAST.

We evaluated V(D)J gene usage in the *de novo* assembled contigs returned by our oracle. We found 31 instances where our oracle failed to return a correct immunoglobulin sequence in contrast to BASIC (Fig. 1C; Column AK, Supplementary Table S1). At the same time, there were only 4/12 instances where our oracle successfully returned a correct sequence when BASIC could not. In sum, our oracle failed to return a correctly assembled sequence among Trinity’s contigs for 42/382 (or 11%) of the immunoglobulin sequences (Supplementary Table S2). Taken together, our analysis demonstrates the importance of our guided, anchor-based approach.

## 3 Discussion

The BCR is generated through an immunological process known as affinity maturation and is ultimately secreted in soluble form as antibodies. During this antibody affinity maturation process, somatic rearrangements and mutations are introduced into the BCR to improve antigen recognition. We have introduced a novel algorithm, BASIC, which enables investigators to assemble BCR sequences from scRNA-seq data and study B-cell repertoire at single-cell resolution. We have experimentally validated sequences assembled by BASIC, and demonstrate overall high accuracy and in particular the superiority of an anchor-guided assembly strategy over a purely *de novo-*based approach. For the first time, BASIC can help couple gene expression information from scRNA-seq with immune repertoire, and facilitate studies between B-cell receptor features, clonality, differentiation and transcriptional programming at single-cell resolution.

## Supplementary Material

Supplementary Data
