# Supplementary material for: BASIC: BCR assembly from single cells
Source: Bioinformatics. 2016 Oct 2;33(3):425–7. doi: 10.1093/bioinformatics/btw631 (PMC5408917; doi:10.1093/bioinformatics/btw631)
Supplement: Supplementary Data [file btw631_supp.zip › BASIC_Supplementary_Material.docx]

| Genome analysis  BASIC: BCR assembly from single cells  Stefan Canzar^1,#^, Karlynn E. Neu^2,#^, Qingming Tang^1^, Patrick C. Wilson^2^ and Aly A. Khan^1,*^  ^1^Toyota Technological Institute at Chicago, Chicago, IL 60637, USA, ^2^Committee on Immunology, The Knapp Center of Lupus and Immunology Research, The University of Chicago, Chicago, IL 60637, USA  *To whom correspondence should be addressed. #Equal contribution.  **Contact:** aakhan@ttic.edu |
| --- |

**Supplementary Material**

1. Supplementary Methods
2. Supplementary Figure 1
3. Supplementary Figure 2
4. Supplementary Table 1
5. Supplementary Table 2
6. Supplementary Data 1 (Sanger sequencing Results)
7. Supplementary Data 2 (BASIC assembly Results)
8. Supplementary Data 2 (Trinity assembly Results)

# Supplementary Methods

We developed BASIC to determine the full-length sequence of the heavy and the light chains in the BCR of a single B cell from scRNA-seq. Briefly, BASIC performs semi-*de novo* assembly in two stages (Supplementary Figure 1): Stage 1, BASIC uses known constant and variable regions to identify anchor sequences; Stage 2, BASIC uses these anchors to guide the *de novo* assembly of the BCR.

Stage 1: The BASIC software uses a pre-compiled database of known variable (IGHV, IGKV, IGLV) and constant (IGHC, IGKC, IGLC) region sequences in human from IMGT (http://www.imgt.org). The database was indexed using Bowtie2 (Langmead & Salzberg, 2012) into four distinct files that corresponded to different components (IGHV; variable heavy chain, IGHC; constant heavy chain, IGK/LV; variable light chain, IGK/LC; constant light chain). Second, BASIC calls Bowtie2 in order to align scRNA-seq reads from a single B cell to each of the four component index files. Third, for each component, BASIC identifies a short sequence window containing the highest number of aligned reads. These four sequences served as anchors to guide the assembly stage. As expected for anchors in the variable regions, sequences typically mapped to the non-complementarity determining regions (non-CDR). Lastly, we filtered all remaining low-complexity reads for which at least half of the read sequence was composed of a single nucleotide.

Stage 2: Next, BASIC performs *de novo* assembly to stitch together the anchor sequences in the heavy and light chains. We assume a sequence may overlap either with the forward sequence or the reverse complement sequence of another read. Two reads overlap if the prefix of one sequence equals the suffix of the other sequence (or vice versa). Briefly, BASIC extends each anchor iteratively in the 3' direction (one read at a time) until there is either no overlapping read or a repeat is found. Then, each anchor is extended in the 5’ direction in the same way. For each chain, BASIC reports a single sequence if the extended sequence from the variable region anchor is equal to the extended sequence from the constant region anchor. However, if the two sequences did not match, BASIC reports both an extended variable region contig and an extended constant region contig, separately. Two contigs will result, for example, when there is not enough sequencing read coverage spanning the entire BCR.

To efficiently extend the anchor sequences we developed a greedy minimum entropy approach. In each iteration, starting with the anchor sequence, we identified all overlapping reads for a given direction. This can be seen as generating a star graph with one internal node (current sequence) and edges to all possible reads overlapping the current sequence. In contrast to other assembly graphs that use a fixed k-mer size, we conceptually explore a graph structure that connects nodes by an edge if the corresponding read sequences overlap by any length k. In that way, we are able to handle uneven sequencing coverage of the BCR transcript, which is common in scRNA-seq.

More formally, we define $r_{i}^{k}$ as the $i$th read with an overlap of length $k$ and $c\left( r_{i}^{k} \right)\in\mathbb{Z}^{+}$ as the read count. Importantly, reads $r_{i}^{k}$ and $r_{j\neq i}^{k}$ with the same $k$ length that both overlap the current sequence may differ due to substitution errors in the non-overlapping portion. We seek to select an overlapping read that can extend the current sequence and whose selection is supported by a high read count and low uncertainty in the non-overlapping portion. Critically, we can measure uncertainty as a function of the number of different reads with the same $k$ length overlap.

First, we determined the optimal $k$ length overlap from which to choose a read and extend the current sequence. We selected a length $k^{*}$ with the ‘minimum amount of uncertainty' (minimal entropy) based on the number of different reads with the same $k$:

$$k^{*}=\arg\min_{k} \left[ -\sum_{i} P\left( r_{i}^{k} \right)\text{log}_{2}(P\left( r_{i}^{k} \right)) \right]\text{, where} P\left( r_{i}^{k} \right)=\frac{c\left( r_{i}^{k} \right)}{\alpha+\sum_{j} c\left( r_{j}^{k} \right)}$$

We also added a small bias term $\alpha\in[0,1]$ in $P\left( r_{i}^{k} \right)$ so that higher read counts are favored when there is *a priori* an equal proportion of reads in different $k$. Second, we sought a single representative read $r_{i}^{k}$ based on $i^{*}$ the largest read count or the largest probability of occurring for a given length $k$:

$$i^{*}=\arg\max_{i} P\left( r_{i}^{k}|k \right)$$

Finally, the representative read was merged with the current sequence into a single longer sequence. This process was repeated until there was either no overlapping reads or a repeat was found. The running time of the algorithm mostly depends on the length of the sequencing reads and the number of reads. Using two CPU cores, the average wall clock run time for semi-*de novo* assembly of both heavy and light chains was 30 minutes per cell (including alignment time). We note that efficient data structures such as suffix trees or FM indexes can allow different trade-offs in speed and memory for a more comprehensive exploration of an overlap graph during *de novo* assembly in Stage 2.

# Supplementary Figure 1

**Supplementary Figure 1)** Schematic illustration of steps in the algorithm that select and extend the sequencing reads from scRNA-seq data. Supplementary Figure 2 contains a box-plot of entropy values as a function of k for PW1_A1 sample during heavy and light chain assembly.

# Supplementary Figure 2

**Supplementary Figure 2)** Box-plot of entropy values as a function of k for PW1_A1 sample during heavy and light chain assembly. Typically, there is higher certainty in how to extend a contig when there is greater overlap between a read and the current sequence. Entropy values scaled [0,1].

# Supplementary Table 1

BASIC_Supplementary_Table_1: IgBlast results for Primer Cocktail and Primer Specific PCR Sanger sequencing in addition to BASIC assembled sequences and Trinity's best match contigs.

Details:

- Gene identification done with IgBlast.
- PW1 samples are single-end sequenced; PW2 and PW3 are paired-end sequenced.
- The 'Alternative' genes have identical percent identity to the BCR sequence and all have equal probably of being correct.
- Highlighted samples are samples available through EBI; other samples are available upon request.
- Samples in red are either: mismatches from ground truth or samples where no BCR sequence was identified (both a V and a J gene ID was required to be included in analysis).
- Blue are the situations where BASICs predicted BCR was present in the BCR contigs provided by Trinity but this BCR does not match the PCR obtained ground truth.

# Supplementary Table 2

Summary of errors for BCR assembly from Trinity:

| Categories of Trinity Errors | # errors |
| --- | --- |
| Incomplete BCR assembly | 21 |
| No BCR assembly | 10 |
| BCR assembled, incorrect genes | 6 |
| BCR assembled, incorrect genes *(same as BASIC)* | 5 |
| **Failure to assemble BCR** | **31** |
| **BCR assembled but incorrect** | **11** |
| **Total errors** | **42** |

# Supplementary Data 1 (Sanger sequencing results)

Fasta file available for EBI hosted samples: http://ttic.uchicago.edu/~aakhan/BASIC/data/sanger.fasta

# Supplementary Data 2 (BASIC assembly results)

Fasta file available for EBI hosted samples: http://ttic.uchicago.edu/~aakhan/BASIC/data/BASIC.fasta

# Supplementary Data 3 (Trinity B1 assembly results)

Best contig reported by Trinity that mapped to B1 heavy chain

http://ttic.uchicago.edu/~aakhan/BASIC/data/Trinity/B1_TRINITY.fasta
